# A Smartphone Physical Activity App for Patients in Alcohol Treatment: Single-Arm Feasibility Trial

**DOI:** 10.2196/35926

**Published:** 2022-10-19

**Authors:** Ana M Abrantes, Lidia Z Meshesha, Claire E Blevins, Cynthia L Battle, Clifford Lindsay, Eliza Marsh, Sage Feltus, Matthew Buman, Emmanuel Agu, Michael Stein

**Affiliations:** 1 Butler Hospital Providence, RI United States; 2 Alpert Medical School of Brown University Providence, RI United States; 3 Department of Psychology University of Central Florida Orlando, FL United States; 4 UMass Chan Medical School Worcester, MA United States; 5 Arizona State University Tempe, AZ United States; 6 Worcester Polytechnic Institute Worcester, MA United States; 7 Boston University Boston, MA United States

**Keywords:** alcohol use disorder, AUD, physical activity, smartphone app, Fitbit, feasibility study, mobile phone

## Abstract

**Background:**

Alcohol use disorder (AUD) is a significant public health concern worldwide. Alcohol consumption is a leading cause of death in the United States and has a significant negative impact on individuals and society. Relapse following treatment is common, and adjunct intervention approaches to improve alcohol outcomes during early recovery continue to be critical. Interventions focused on increasing physical activity (PA) may improve AUD treatment outcomes. Given the ubiquity of smartphones and activity trackers, integrating this technology into a mobile app may be a feasible, acceptable, and scalable approach for increasing PA in individuals with AUD.

**Objective:**

This study aims to test the Fit&Sober app developed for patients with AUD. The goals of the app were to facilitate self-monitoring of PA engagement and daily mood and alcohol cravings, increase awareness of immediate benefits of PA on mood and cravings, encourage setting and adjusting PA goals, provide resources and increase knowledge for increasing PA, and serve as a resource for alcohol relapse prevention strategies.

**Methods:**

To preliminarily test the Fit&Sober app, we conducted an open pilot trial of patients with AUD in early recovery (N=22; 13/22, 59% women; mean age 43.6, SD 11.6 years). At the time of hospital admission, participants drank 72% of the days in the last 3 months, averaging 9 drinks per drinking day. The extent to which the Fit&Sober app was feasible and acceptable among patients with AUD during early recovery was examined. Changes in alcohol consumption, PA, anxiety, depression, alcohol craving, and quality of life were also examined after 12 weeks of app use.

**Results:**

Participants reported high levels of satisfaction with the Fit&Sober app. App metadata suggested that participants were still using the app approximately 2.5 days per week by the end of the intervention. Pre-post analyses revealed small-to-moderate effects on increase in PA, from a mean of 5784 (SD 2511) steps per day at baseline to 7236 (SD 3130) steps per day at 12 weeks (Cohen *d*=0.35). Moderate-to-large effects were observed for increases in percentage of abstinent days (Cohen *d*=2.17) and quality of life (Cohen *d*=0.58) as well as decreases in anxiety (Cohen *d*=−0.71) and depression symptoms (Cohen *d*=−0.58).

**Conclusions:**

The Fit&Sober app is an acceptable and feasible approach for increasing PA in patients with AUD during early recovery. A future randomized controlled trial is necessary to determine the efficacy of the Fit&Sober app for long-term maintenance of PA, ancillary mental health, and alcohol outcomes. If the efficacy of the Fit&Sober app could be established, patients with AUD would have a valuable adjunct to traditional alcohol treatment that can be delivered in any setting and at any time, thereby improving the overall health and well-being of this population.

**Trial Registration:**

ClinicalTrials.gov NCT02958280; https://www.clinicaltrials.gov/ct2/show/NCT02958280

## Introduction

### Background

Alcohol use disorder (AUD) is a public health problem that has a significant negative impact on both individuals and society [1,2]. Data from the US National Epidemiological Survey on Alcohol and Related Conditions III indicate a lifetime prevalence rate of 29.1% for the Diagnostic and Statistical Manual of Mental Disorders, Fifth Edition, diagnoses of AUD [3]. Alcohol consumption is a leading cause of death in the United States and is associated with a significant economic burden [4,5]. Despite the existing treatments for AUD, relapse rates in the first year following treatment are high [6-8]. Therefore, adjunct intervention approaches to improve alcohol outcomes during early recovery are critical.

Over the last 2 decades, physical exercise has emerged as an adjunct intervention for alcohol treatment. There are a number of biological mechanisms that can explain the potential effect of exercise on alcohol treatment outcomes. For example, exercise may normalize disrupted dopaminergic signaling in patients with AUD [9]. Through its influence on these neural reward pathways, exercise may serve as a competing reinforcer that diminishes alcohol use [10]. Furthermore, decreases in attentional bias to alcohol cues after bouts of exercise may acutely decrease alcohol cravings [11]. Another potential mechanism involves the effects of exercise on cognitive functioning, particularly executive functioning, both in the acute and long term [12,13]. Improved neurocognitive functioning can aid in treatment retention and decision-making, both of which affect alcohol relapse [14].

Owing to these potential mechanisms, there has been increased attention toward the development and testing of physical activity (PA) interventions for individuals with AUD. Indeed, this study has demonstrated that increasing PA during early recovery from AUD can be beneficial for a number of important reasons. First, patients undergoing AUD treatment express a strong interest in increasing PA as a means of helping to support their recovery [15,16]. Second, patients with AUD have significant alcohol-related health concerns (eg, hypertension, diabetes, and liver disease [17,18]) that are exacerbated by low levels of PA and ameliorated with increase in PA [19,20]. Third, negative affect associated with comorbid internalizing disorders and stressful life events is common among patients with AUD and increases the risk of self-medication and drinking to cope [21]. Both acute bouts of PA and long-term engagement in a PA program have been associated with decreases in negative affect, depression, and anxiety [22,23], including in patients with AUD [24,25]. Finally, although empirical support for the effect of PA interventions on drinking outcomes in patients with AUD has been limited [26], evidence for the acute effect of bouts of PA, even those of relatively brief durations, on reductions in alcohol cravings has been demonstrated across a number of studies [11,27]. Therefore, strategies to increase PA in early recovery are likely to be well received and potentially helpful for patients’ physical and mental health, as well as potentially promoting abstinence.

Although prior studies examining PA as an intervention approach for individuals in AUD treatment demonstrated promising outcomes in terms of cardiorespiratory and psychological functioning, and in a few cases, alcohol outcomes [26], there are also limitations associated with this existing work. Many of these studies were conducted more than 2 decades ago, and the interventions were conducted primarily in residential treatment facilities, although the majority of AUD treatments were delivered in outpatient settings [28]. Most of the interventions tested involved supervised, structured exercise programs without consideration of the participants’ PA preferences. Although these types of approaches have their advantages (eg, social support), scheduling conflicts, transportation difficulties, and a restricted range of PA type may impact PA adherence. High dropout rates are common [26]. Finally, few studies have incorporated theoretically informed cognitive or behavioral features to increase motivation for PA [29]. Therefore, the development of PA interventions that can address these key limitations may lead to better outcomes in patients with AUD.

Using technology such as activity monitors and smartphones to support PA interventions has numerous advantages. For example, self-monitoring of PA is one of the most effective strategies for increasing PA [30] but is burdensome for individuals. Activity monitors and smartphone apps may ease this burden (ie, people usually have their phones with them) [31] in an efficient, interactive, and tailored manner [32]. Activity monitors and smartphone apps provide device-based PA feedback that can be used to produce individualized goal setting. Another advantage of using mobile apps to deliver PA interventions is the decreased need for formal training of providers on how to conduct PA counseling in individuals with AUD [33]. Furthermore, as smartphones are owned by over 85% of the population across most demographics [34], smartphone apps are a potentially cost-effective approach that can reach a wide number of individuals with AUD, with the ability to convey standardized therapeutic information within the app.

Although many smartphone-based PA apps are available, very few are theoretically informed or have been empirically evaluated [35,36] and none, beyond this study, have targeted a population with AUD. Most people are motivated to exercise for health enhancement, weight loss, and appearance [37]. However, long-term engagement in PA continues to be a significant public health challenge, including for those with AUD [26]. Social cognitive theory [38] posits that behavior is increased or sustained when it is immediately reinforced, and self-determination theory [39] identifies intrinsic goals and motivation as key for long-term adherence to PA. Indeed, personally meaningful goals (rather than culturally or societally driven expectations of exercise) are critical for sustaining behavior change [40]. A PA smartphone app may help patients with AUD in early recovery to identify and support unique intrinsic sobriety-related goals and motivations that, if integrated with their PA goals, could lead to the adoption and maintenance of an exercise program. For example, by using a mobile app that facilitates self-monitoring and provides feedback, patients with AUD could increase their awareness of the immediate benefits of PA bouts on negative affect and alcohol cravings [24] and, in turn, be more likely to persist with PA.

### Objectives

The purpose of this study was to determine the feasibility and acceptability of a recently developed PA smartphone app called Fit&Sober for patients with AUD. To do so, we conducted an open trial in which the Fit&Sober app was pilot-tested for 12 weeks in a small sample of individuals with AUD in early recovery (N=22). The details on the formative work conducted in developing the Fit&Sober app are available in the study by Abrantes et al [41]. We hypothesized that participants would report that using the Fit&Sober app was feasible and acceptable, while also demonstrating increase in PA and quality of life and decrease in alcohol consumption, anxiety, depression, and alcohol craving after 12 weeks of app use.

## Methods

### Procedure and Study Design

The Alcohol and Drug Partial (ADP) hospitalization program runs every day of the week from 9 AM to 3:30 PM. ADP is an abstinence-based, relapse prevention program focused on increasing cognitive behavioral skills for sobriety. The patients participated in daily group and individual therapy, received medication management, and were discharged with aftercare plans in place. The length of stay ranged from 5 to 10 days. Upon treatment admission, the patients’ medical records were screened, and those who met the study criteria were provided with brief information about the study. Interested participants underwent a brief screen (5-10 minutes) to determine their PA levels and smartphone ownership. If eligible, the participants were scheduled for a more comprehensive baseline assessment (approximately 90 minutes) on the following day. Informed consent was obtained, and assessment measures to confirm eligibility were conducted at the baseline appointment. The study physician reviewed the patient’s medical history and results of a routine physical examination conducted during partial hospitalization. Upon receiving medical clearance to exercise, participants were enrolled into the open pilot trial and scheduled for an app orientation session. All procedures were performed within 5 to 10 days of partial hospitalization. Participants were contacted at 2, 6, and 12 weeks after discharge from ADP hospitalization to provide feedback on their use of the Fit&Sober app and to complete clinical outcome measures (at 12 weeks only).

### Participants

The inclusion and exclusion criteria for the study are presented in Textbox 1.

A total of 48 patients in an ADP hospitalization program in the northeast region of the United States were screened for study eligibility from December 2017 to June 2018. Of these, 42% (20/48) of participants did not meet the eligibility criteria for the following reasons: they were too physically active (11/20, 55%), did not own a smartphone (6/20, 30%), were diagnosed with a manic episode in the past 6 months (1/20, 5%), were diagnosed with a moderate or severe substance use disorder (1/20, 5%), or were not medically cleared (1/20, 5%). In addition, 8% (4/48) of participants declined to participate, and 4% (2/48) of participants did not complete all baseline procedures, leaving 22 participants who were fully eligible and enrolled in the study.

**Inclusion and exclusion criteria.**

**Inclusion criteria**

- Aged between 18 and 65 years
- Met the Diagnostic and Statistical Manual of Mental Disorders (DSM), Fifth Edition, criteria for alcohol use disorder as assessed by the Structured Clinical Interview for the DSM-Patient Version
- Reported low physical activity (ie, <90 minutes of moderate-intensity exercise per week for the past 6 months)
- Engaged in alcohol treatment
- Owned a smartphone

**Exclusion criteria**

- Clinical diagnosis from a medical record review of current moderate-severe substance use disorder (except nicotine), anorexia, bulimia nervosa, or mania
- History of psychotic disorder or had current psychotic symptoms
- Endorsed current suicidality or homicidality
- Physical or medical problem that would not allow safe participation in a program of moderate-intensity physical activity
- Currently pregnant or expressed intent to become pregnant during the next 12 weeks

### Ethical Considerations

All procedures performed in the studies involving human participants were in accordance with the ethical standards of the institutional review board of Butler Hospital in Providence, Rhode Island, United States (protocol 1604-003). Informed consent was obtained from all the participants included in the study. This study was registered at ClinicalTrials.gov (NCT02958280).

### Intervention

#### App Orientation Session

In preparation for the app orientation session, the research staff downloaded the Fit&Sober and Fitbit apps onto the participants’ smartphones. The orientation session was conducted by a doctoral-level study clinician and lasted for approximately 30 to 40 minutes. It included brief advice on increasing PA during early recovery, providing an overview and walkthrough of Fit&Sober app components, and connecting a wrist-worn Fitbit device (Charge HR) to the Fit&Sober app. Brief advice provided participants with information on the public health guidelines for PA, the benefits of PA for physical and mental health, as well as sobriety, strategies for getting started, and instruction on gradually increasing PA. Then, participants were guided through the Fit&Sober app’s setup that included entry of their sobriety date, selection of values-driven statements on reasons for increasing PA during early recovery (eg, “Being physically active will help me stay sober”), names of social support for PA and recovery, inputting of initial PA goals (ie, steps per day and minutes per week of PA), and present-moment ratings of mood and alcohol cravings. Finally, with the participants’ permission, their Fitbit accounts were connected to the Fit&Sober app so that their PA data migrated to the Fit&Sober app and populated PA graphs on the Fit&Sober app’s dashboard in real time. Participants were instructed to use the Fit&Sober app daily for the next 12 weeks.

#### Fit&Sober App

The development of the Fit&Sober app was guided by social cognitive theory [38] and self-determination theory [39], and a detailed description of its features and components can be found in the study by Abrantes et al [41], along with specific links between theory concepts and app features. In brief, the goals of the Fit&Sober app were to facilitate self-monitoring of PA engagement and daily mood and alcohol cravings, increase awareness of the immediate benefits of PA on mood and cravings, encourage setting and adjusting PA goals, provide resources and increase knowledge for increasing PA, and serve as a resource for alcohol relapse prevention strategies.

**Figure 1:**
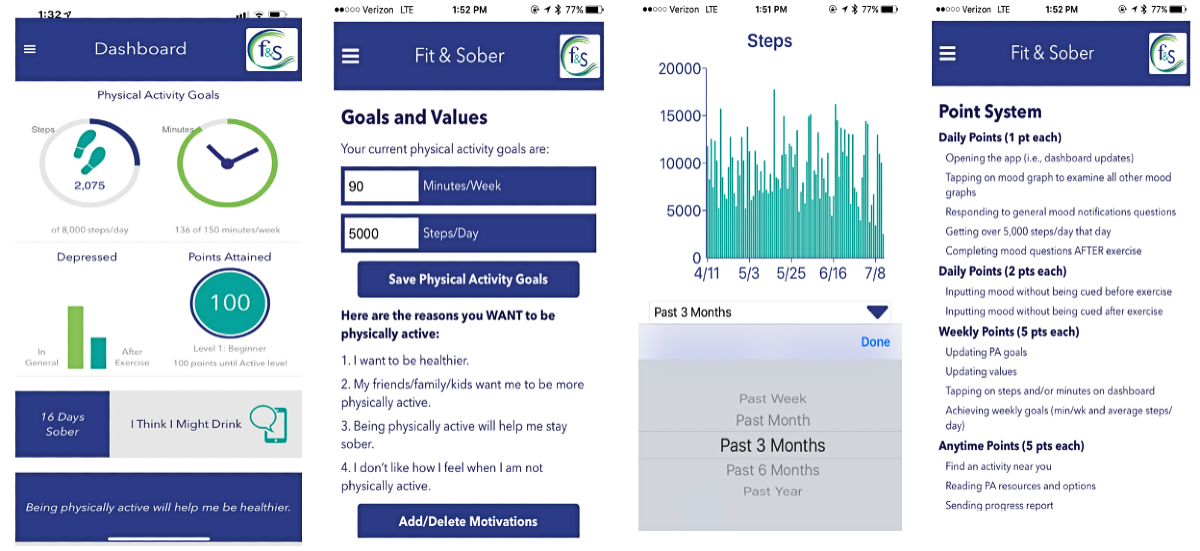
Screenshots of the Fit&Sober app.

Therefore, the Fit&Sober app was designed as a readily accessible tool to aid patients in early alcohol recovery and improve treatment outcomes through increased engagement in PA. To that end, the following functionalities were available in the app: (1) real-time graphical displays of daily progress toward step per day goal and weekly progress toward minutes per week of PA; (2) personalized, values-based messages on reasons for engaging in PA during early recovery that are refreshed each time the user opens the app; (3) daily notifications to input mood and cravings ratings in the morning and after bouts of PA are detected that are then graphically displayed on the dashboard to reflect changes in mood and cravings after engaging in PA; (4) the ability to directly contact social supports for PA and recovery directly from the app (eg, send an SMS text message), when needed; (5) the ability to find local community PA resources by inputting zip codes; (6) alcohol relapse prevention resources that include strategies for managing cravings, a link to the meeting finder on the Alcoholics Anonymous website, and ready access to sobriety support through SMS text messaging or phone call to their identified network of supports; and (7) dashboard view of total days sober, with the ability to change sobriety date. Finally, efforts to support engagement with the Fit&Sober app included gamification and inactivity notices. Specifically, participants were able to collect points as they engaged in the app (eg, completed mood ratings and updated PA goals) and were able to move up in *rank* based on the number of points they collect. Figure 1 presents screenshots of the Fit&Sober app.

### Measures

#### Alcohol Use

The Timeline Follow Back (TLFB) [42] was administered at baseline and at the end of treatment (EOT) to assess the frequency of alcohol use over the previous 90 days. The TLFB uses anchor dates such as holidays to prompt participant recall. Using data from the TLFB, we calculated 2 indices of alcohol use for our analyses: days of alcohol use and percentage of abstinent days.

#### Alcohol Craving

The Penn Alcohol Craving Scale [43] is a 5-item measure designed to assess recent craving. Participants were asked to rate their experiences over the past week on a scale of 0 (low) to 6 (high). In this study, Cronbach *α* reliability coefficients were excellent, with an *α* of .89 at baseline and .98 at EOT.

#### Self-report PA

Consistent with adding exercise as a “vital sign” [44], self-reported levels of PA were assessed by asking participants (1) Over the last 3 months, on average how many days per week did you exercise? and (2) On those days, on average how many minutes per day did you exercise? The weekly time spent exercising was calculated by multiplying these 2 responses.

#### Objectively Measured PA

At baseline, participants wore a GT3x accelerometer (ActiGraph). The duration of accelerometer use was dependent on the timing of study enrollment relative to hospital discharge. Considering the varying length of stay, it was not possible for all participants to wear the accelerometer for the desired 7 days, the gold standard for in vivo accelerometry assessments. For the purposes of this study, baseline step counts were calculated for participants with at least one valid day, defined here as 8 hours per day of accelerometer wear time. Wear time validation was conducted using Actilife Software (ActiGraph, LLC), using the Choi algorithm [45].

#### Depressive Symptoms

Depressive symptoms were assessed using the Center for Epidemiologic Studies Depression Scale-Revised [46]. This scale is a 20-item measure that assesses depressive symptoms on a 4-point scale (0-3). After reverse coding appropriate items, the depressive symptom score was obtained by summing the 20 items, with higher scores indicating higher levels of depressive symptoms (Cronbach *α*=.82 at baseline and Cronbach *α*=.86 at EOT).

#### Anxiety Symptoms

The Generalized Anxiety Disorder-7 [47] was used as a measure of generalized anxiety disorder symptomatology. The Generalized Anxiety Disorder-7 asks participants to rate how often over the last 2 weeks they have experienced 7 symptoms of anxiety on a scale of 0 (not at all) to 3 (nearly every day; *α*=.86 at baseline and *α*=.94 at EOT).

#### Quality of Life

This was measured using the Quality of Life Enjoyment and Satisfaction Questionnaire-Short Form [48]. This questionnaire is a 16-item self-report measure of enjoyment and satisfaction in various domains of life, including physical health, mood, work, leisure, and social activities. Participants rated their satisfaction in the past week on a 5-point scale (1=very poor to 5=very good). In this study, *α* reliability coefficients were excellent (*α*=.95 at baseline and *α*=.96 at EOT).

#### Usability and Acceptability

Participants completed the 8-item Client Satisfaction Questionnaire [49] at EOT that assessed the level of satisfaction with the app and overall program on a scale of 1 to 4, with higher numbers indicating greater satisfaction.

The participants completed the 10-item System Usability Scale (SUS) [50,51] at the 12-week EOT assessment. The range of scores on the SUS was 0 to 100, and scores of ≥70 indicated good usability and satisfaction [52]. We also evaluated the attractiveness and detail of the 8 design features on the app on a scale of 1 (very unattractive or not detailed at all) to 5 (very attractive or very detailed). Participants were then asked to rate the usefulness of the proposed app components on a scale of 1 (not useful at all) to 5 (very useful).

Participants were asked to rate their experience with the Fitbit tracker using the 19-item Participant Experience Questionnaire of Wearable Activity Trackers [53], with items rated on a 5-point Likert scale ranging from 1 (strongly disagree) to 5 (strongly agree). Sample items included, “I found the Fitbit activity tracker to be clear and understandable to use” and “I have the technology necessary to use the Fitbit activity tracker.”

To determine the helpfulness of the specific Fit&Sober app components, participants were asked to rate a series of statements on a scale of 0 (not helpful) to 10 (extremely helpful). This measure contained 13 items and included items such as, “I am able to identify my social support for exercise (eg, someone to exercise with me)” and “I am able to see how my cravings or urges for alcohol change with exercise.”

#### App Use

Participants were asked to estimate how many days per week they used the app and which components of the app they used at 2, 6, and 12 weeks after discharge. In addition, the frequency of days when the Fit&Sober app was opened was collected from the app metadata.

### Data Analysis Plan

We presented descriptive statistics for participant characteristics at baseline, including demographic information, self-reported alcohol use, PA, depression, anxiety, and quality of life. Baseline demographic and clinical characteristics were compared between participants who completed the EOT assessments and those who did not, using chi-square and 2-tailed *t* tests. Descriptive statistics were presented to evaluate the participants' perceptions of acceptability and feasibility. Given the developmental nature of this project, we conducted an open pilot study with a small sample (N=22), although sufficient to provide relatively stable group means for the dependent measures of interest. Therefore, we refrained from conducting significance testing and instead calculated effect sizes [54] in the form of Cohen *d* [55].

## Results

### Participant Characteristics

Participants (N=22) ranged in age from 20 to 61 years, with a mean age of 43.64 (SD 11.57) years, including more than half of the sample being women (13/22, 59%) and most being White (21/22, 95%) individuals. At baseline, participants drank an average of 65.09 (SD 19.79) days in the past 90 days, and when they drank, they averaged 9.2 (SD 3.8) drinks per drinking day. Half of the participants (11/22, 50%) used an iPhone and the other half (11/22, 50%) used an Android phone. At the 3-month follow-up, 86% (19/22) of patients provided data.

### Feasibility and Acceptability

App use was determined by self-report at the 2-, 6-, and 12-week assessments and by examination of the Fit&Sober metadata. Table 1 presents the mean number of days per week that participants reported opening and interacting with the app, as well as the percentage of participants who interacted with specific app features at each time point. Table 2 presents the app metadata on the percentage of the sample who used the app (ie, opening the app on a given day) and the average number of days per week of use across the 12-week intervention period. On average, at the end of the intervention period, participants self-reported using the app 4.33 (SD 2.72) days per week. In comparison, the Fit&Sober app metadata suggested that participants used the app, defined as opening the app, 2.55 (SD 1.68) days per week. At the EOT, on average, the SUS total score was 74.64 (SD 14.90) for the Fit&Sober app. Further, the app’s average attractiveness was 3.20 (SD 1.16), and its usefulness was 3.11 (SD 1.07) on a scale of 1 to 5. Satisfaction ratings on the Client Satisfaction Questionnaire were generally high, with an average of 3.40 (SD 0.43) for the full scale (on a 1-4 scale). Individual item-level mean ratings were as follows: quality of program 3.44 (SD 0.51), the kind of program participant wanted 3.31 (SD 0.60), program meeting participant needs 3.13 (SD 0.81), recommend to a friend 3.63 (SD 0.62), amount of help received 3.44 (SD 0.89), helped with increasing PA 3.44 (SD 0.63), overall satisfaction 3.38 (SD 0.81), would come back to program in the future 3.44 (SD 0.51), and a supplemental question of whether PA helped with alcohol recovery 3.31 (SD 0.87).

Overall, participants found the app helpful, 6.26 (SD 2.11) on a 1 to 10 scale. Table 3 details the item-level ratings of how helpful the participants found the various components of the app. The components that received a high rating of ≥7 were keeping track of their PA, capability to see the number of sober days, observe mood change with PA, and the app’s ability to communicate with the Fitbit tracker. The components that received a lower rating of ≤5 were finding social support for PA, sobriety, and seeing motivational quotes every day. Finally, participants wore the Fitbit for an average of 9.5 (SD 3.7) weeks (ie, 79%) of the intervention period) and reported being generally satisfied with the tracker, with an average rating of 4.04 (SD 1.16) at the EOT.

The mean of number of days of valid accelerometer wear time was 2.4 (SD 1.4). Accelerometry data collected at baseline were available for 95% (21/22) of participants. The baseline wear time for this sample (N=22) was as follows: 1 day (4/22, 18%), 2 days (9/22, 41%), 3 days (4/22, 18%), 4 days (2/22, 9%), 5 days (1/22, 5%), and 6 days (1/22, 5%). Daily step counts were collected throughout the 12-week intervention objectively via the Fitbit, with average daily step counts calculated for participants who had at least 8 weeks of Fitbit data, with Fitbit devices worn at least 8 hours per day (18/22, 82%).

**Table 1:** Self-reported use of the Fit&Sober app.

App use features		Weeks 1 and 2 (n=15)	Weeks 3-6 (n=14)	Weeks 7-12 (n=19)
Opened the app (days per week), mean (SD)		5.33 (1.95)	4.33 (2.6)	4.21 (2.7)
Interacted with the app^a^ (days per week), mean (SD)		5.04 (2.42)	3.95 (2.81)	3.63 (2.89)
**Sample who interacted with each of the following Fit&Sober app features, n (%)**				
	Updated PA^b^ goals	10 (67)	8 (57)	5 (26)
	Responded to notifications the app sent	12 (79)	9 (62)	13 (68)
	Entered mood ratings after a bout of PA	13 (87)	10 (71)	9 (47)
	Reviewed mood ratings graphs	9 (60)	11 (77)	11 (58)
	Updated social supports for PA and sobriety	1 (7)	0 (0)	0 (0)
	Updated sobriety date	0 (0)	2 (14)	0 (0)
	Updated reasons for PA	1 (7)	1 (7)	1 (5)
	Reviewed the alcohol relapse prevention strategies	5 (27)	5 (36)	5 (26)
	Looked for ideas for increasing PA	6 (40)	7 (50)	4 (21)

^a^Do something on the app.

^b^PA: physical activity.

**Table 2:** Objective app use from the Fit&Sober app metadata.

Intervention week	1	2	3	4	5	6	7	8	9	10	11	12
Sample who used the app, n (%)	18 (82)	17 (77)	18 (82)	16 (73)	14 (64)	14 (64)	14 (64)	16 (73)	12 (55)	11 (50)	12 (55)	10 (45)
App use (days per week), mean (SD)	4.4 (1.5)	3.7 (2.5)	3.4 (2.5)	2.4 (2.1)	1.9 (1.9)	2.7 (2.3)	2.8 (2.4)	2.8 (2.5)	1.8 (2.1)	1.8 (2.2)	1.7 (1.8)	1.6 (2.2)

**Table 3:** Item-level ratings of the self-reported helpfulness of various components of the Fit&Sober app^a^.

App feature	Value, mean (SD)
1. I am able to set my own exercise goals	6.29 (2.89)
2. I am able to see my progress toward achieving my exercise goal	6.59 (2.85)
3. I am able to track my physical activity	7.29 (2.57)
4. I am able to keep track of how many days I have been sober	8.59 (2.00)
5. I am able to identify my social supports for exercise	4.71 (3.35)
6. I am able to identify my social supports for sobriety	4.59 (3.39)
7. I am able to identify my values and the reasons I am increasing my physical activity	6.06 (3.09)
8. I am able to see how my mood changes with exercise	7.18 (2.86)
9. I am able to see how my cravings or urges for alcohol change with exercise	6.71 (2.87)
10. I am able to view motivational quotes every day	4.88 (3.44)
11. I am able to receive specific advice if I feel an urge to drink	5.24 (3.47)
12. I am able to view resources and ideas for ways I can be more physically active	5.47 (3.12)
13. The app is able to communicate with the Fitbit tracker	7.82 (2.30)

^a^The range of items is 1 to 10 with higher ratings indicating greater helpfulness.

### PA Outcomes

**Figure 2:**
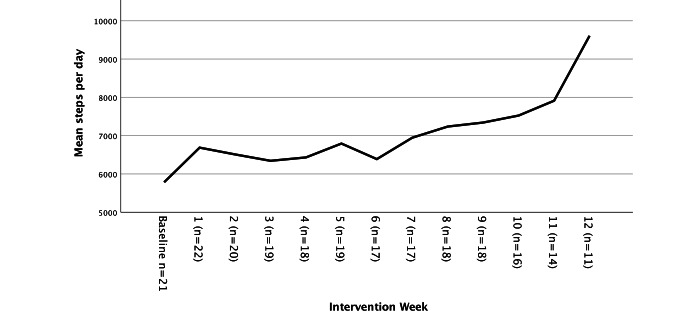
Steps per day (Accelerometer at baseline and Fitbit during the 12-week intervention.

PA assessments included self-reported average minutes per week of exercise (assessed with the Exercise as a Vital Sign questions) over the last 3 months, 7-day accelerometry-derived steps per day, and daily steps per day from the Fitbit during the 12 weeks of the intervention. Baseline steps per day collected from wearing the GT3x accelerometer averaged 5783 (SD=2511) steps per day. Among the participants who wore Fitbit for at least 8 weeks (18/22, 82%), the average steps per day over the course of the 12-week intervention was 7236 (SD 3130). Self-report of average minutes per week of exercise at baseline was 76.13 (SD 124.11) and at the EOT was 160.82 (SD 149.76): a moderate effect of increase in self-reported PA (Cohen *d*=0.65, 95% CI 0.11-1.16). Self-reported PA over the 12-week intervention and the average, objective measurement of steps per day on the Fitbit during the intervention were highly correlated, although the 95% CI was large (*r*=0.51, 95% CI −0.03 to 0.82). Table 4 presents the means and effect sizes, and Figure 2 presents the changes in steps per day over the course of each week of the intervention.

**Table 4:** Intervention outcome and effect sizes.

Intervention outcomes		Baseline (n=22), mean (SD)	End of treatment (n=19), mean (SD)	Cohen *d* (95% CI)
**Alcohol outcomes**				
	Number of days drinking alcohol in the preceding 90 days	65.09 (19.79)	7.47 (19.92)	−2.27 (−3.12 to −1.40)
	Percentage of abstinent days	27.68 (21.99)	91.10 (23.72)	2.17 (1.33 to 2.99)
	Alcohol craving	13.41 (7.01)	9.56 (8.90)	−0.36 (−0.86 to 0.16)
**Mental health outcomes**				
	Anxiety (GAD-7^a^)	13.86 (4.73)	9.31 (7.14)	−0.71 (−1.25 to −0.15)
	Depression (CES-D^b^)	29.09 (9.28)	25.19 (11.81)	−0.58 (−1.10 to −0.40)
	Quality of life	38.47 (12.95)	44.54 (15.33)	0.58 (−0.21 to 1.16)
**PA^c^ outcomes**				
	Self-reported minutes of PA during the 12-week intervention	76.13 (124.11)	160.82 (149.76)	0.65 (0.11 to 1.16)
	Objectively measured steps per day^d^	5784 (2511)	7236 (3130)	0.35 (−0.15 to 0.83)

^a^GAD-7: Generalized Anxiety Disorder-7.

^b^CES-D: Center for Epidemiologic Studies Depression Scale-Revised.

^c^PA: physical activity.

^d^Accelerometer at baseline; average steps per day over 12-weeks with Fitbit (among participants with 8 weeks of Fitbit data; n=18).

### Alcohol Outcomes

Among participants who completed the EOT assessment (n=19), 10 (53%) reported complete abstinence over the course of the 12-week intervention. Among the 9 individuals who consumed alcohol, 7 (78%) did so for 6 days or fewer, 1 (11%) consumed alcohol for 47 days, and 1 (11%) consumed alcohol on 77 days in the past 90 days. Overall, the mean reduction in percentage of abstinent days was a Cohen *d* of 2.77. In addition, participants demonstrated lower levels of alcohol craving at EOT (mean 56, SD 8.90) than at baseline (mean 13.41, SD 7.01, Cohen *d*=0.48). Table 4 presents the means and effect sizes.

### Mental Health Outcomes

Participants who completed the EOT assessment reported decrease in anxiety (Cohen *d*=−0.71, 95% CI −1.25 to −0.15) and depressive symptoms (Cohen *d*=−0.58, 95% CI −1.10 to −0.40) from baseline to the EOT. There were also improvements in the quality-of-life scores (Cohen *d*=0.58, 95% CI −0.21 to 1.16).

## Discussion

### Principal Findings

This open pilot trial examined the use of Fit&Sober, a theoretically informed PA smartphone app, targeted toward patients with AUD, in early recovery. The results of this study show high levels of feasibility and acceptability of the Fit&Sober app over a 12-week period. In addition, large effect sizes were observed for changes in alcohol outcomes, small-to-moderate effects for increases in PA and quality of life, and reductions in anxiety and depression from baseline to the EOT. These findings are promising and suggest that future research to determine the efficacy of the Fit&Sober app compared with a control condition is warranted.

### Comparison With Prior Work

Despite the high ratings on measures of app satisfaction, usability, and helpfulness, app use declined over time, such that fewer than half of the participants were using it at the end of the 12-week intervention period. However, these levels of app use are quite comparable and, in some cases, more favorable than what has been reported in previous studies on both PA and alcohol apps. For example, in testing a well-designed, theoretically informed smartphone app for increasing PA and decreasing sedentary behavior, Direito et al [56] found that participants opened the app an average of 11 days during the entire 8-week intervention. In addition, over the course of 5 months, use of 3 different PA apps showed declines ranging from 45% to 75% in use [57]. Similarly, among at-risk drinkers who were interested in reducing their alcohol consumption, 50% had stopped using an app by the end of the first month [58]. In another study of a Veteran population engaged in alcohol misuse, an alcohol self-management app was used by 96% of the sample in week 1, which decreased to 55% by week 4 [59] Indeed, long-term app use is quite low for health apps—the vast majority of health app users stop using the app after 10 visits and 26% use it only once after downloading it [60]. Our results also suggest that participants may self-report higher use than actual use. Given the social desirability of reporting greater app use observed in our study, future research should consider including objective indicators of app use, such as app metadata.

Although app use consistently wanes over time, it appears that the extent of app use may not be related to either increase in PA or decrease in drinking [61,62]. In other words, even when marked decreases in app use were observed, significant increase in PA and decrease in drinking behaviors also occurred [56,59,63]. Thus, some individuals may be able to increase their PA and sustain this change over time with only a few weeks of app use. In future studies, typologies of user engagement with mobile health (mHealth) apps can be identified and then examined in relation to changes in the desired behavioral outcome (eg, PA or drinking), and the utility of these apps in changing and maintaining specific behaviors can be better determined.

The participants in this study demonstrated a 25% increase in objectively determined steps per day (approximately 1400 steps per day from baseline). Increase of 26.9% in steps per day have been shown to result in significant improvements in health, including decreased blood pressure [64]. In a recent systematic review of PA smartphone apps, there was a mean increase of 476 steps per day in those assigned to the app conditions [65]. Therefore, the observed increase in our study are consistent with those of prior research. However, our baseline accelerometer-determined steps per day was limited by less-than-optimal wear time. Indeed, it was challenging to collect a full 7 days of accelerometer wear time, the gold standard in PA research, given the varied and short duration of partial hospitalization admissions and the goal of orienting patients to the app before their discharge from treatment. However, adherence to wearing the Fitbit tracker was good and similar to other patient populations (eg, 39.6%-85.7% in a systematic review of adherence to PA monitoring devices in adults with cardiovascular disease [66]). Given that Fitbit devices are valid for the measurement of steps [67], are feasibly worn for long durations, and considered satisfactory by users, future studies may consider their use for objectively determining long-term engagement in PA, which has, to date, been limited to self-report.

Certain features of the app were used by a greater number of participants than the other features. Specifically, updating PA goals, monitoring changes in mood, and responding to notifications were more popular than updating social support, sobriety dates, and updating reasons for being physically active. Daily app notifications were targeted toward reminding users to self-monitor mood and cravings and update PA goals, which correspond to the features most used by participants. As such, sending notifications to users is an effective strategy for increasing engagement with specific features of the app. Future studies may benefit from the inclusion of innovative designs that allow for microrandomization messaging and notifications (ie, randomizing messages or notifications at the daily level) to identify those most likely to lead to increases in app engagement and behavior change [68].

The last decade has witnessed an increase in the number of smartphone apps targeting individuals with AUD [69]. The acceptability of smartphone apps among at-risk drinkers is high, but their effect on changes in drinking outcomes has been mixed [70]. Common features of these apps include tracking of drinking, alcohol-related consequences, and locating 12-step meetings and treatment programs [69]. Several of the Fit&Sober app features were directly relevant to alcohol recovery including tracking sobriety date and alcohol cravings, locating 12-step meetings, identifying sober supports, and strategies for preventing relapse. Integrating these alcohol-specific features into an app designed to increase PA is a novel approach that may provide an opportunity to synergistically impact multiple health behavior changes. However, PA apps that also include dietary components are not as effective at increasing PA as those focusing only on PA [65]. Therefore, future research that can test the effectiveness of specific combinations of app components may be necessary to optimize app use.

### Strengths and Limitations

This study has several strengths. Our ability to collect objective metadata from app use allowed us to identify the most used app features. As mHealth apps are typically multicomponent, data regarding the usability of these features are critical for the continued optimization of the apps. In addition, we objectively measured PA outcomes. Indeed, overreliance on self-reported PA has been a limitation of prior PA studies with individuals with AUD. Our study demonstrated the feasibility of these passive data collection modalities and, in turn, increased the methodological rigor of this study.

There are also several limitations that merit further discussion. First, this was a small, open pilot study designed to determine the feasibility of the Fit&Sober app during early alcohol recovery. Although increases in PA and quality of life and decreases in anxiety and depression were observed, these effects cannot be attributed to the Fit&Sober app. It is possible that simply being in early recovery and engaging in these related activities (eg, attendance at 12-step meetings, therapy, and avoiding triggers) would lead to the same outcomes. Therefore, a future randomized controlled trial that accounts for these factors is necessary. Second, the sample lacked racial and ethnic heterogeneity. It is important to understand the feasibility and acceptability of the Fit&Sober app across a more diverse sample of patients. Third, the baseline accelerometry measurement of steps per day must be interpreted with caution, given the limited device wear time. The cost-benefit relationship of whether to delay a PA intervention to collect a rigorous assessment of objective PA measurement for individuals who could most benefit from immediately increasing PA (owing to higher levels of craving in the early periods of abstinence) needs to be examined in future research. Finally, although the 12-week duration of the intervention maps onto the first 90 days of recovery being the highest risk for relapse, any future study on the maintenance of PA would require longer study durations.

### Conclusions

Individuals receiving treatment for AUD were willing to participate in an open pilot trial on the feasibility and acceptability of using a PA smartphone app tailored for early alcohol recovery. Participants used the app at rates consistent with other PA and AUD apps. They reported that utilizing the Fit&Sober app was helpful in increasing PA during the early stages of alcohol recovery. The observed increase in PA in this sample approximate those associated with significant improvements in physical health indicators, a critical finding, given the significant concomitant physical health problems experienced by individuals with AUD. Participants most often used the app to self-monitor PA goals, mood, and alcohol cravings, suggesting that these should be key features of any future efforts to optimize the Fit&Sober app or the development of other mHealth interventions for this population. Within-person changes in mental health and drinking outcomes were promising, although an important next step will be to conduct a randomized controlled trial to determine the efficacy of the Fit&Sober app in improving these alcohol treatment outcomes. In conclusion, if the efficacy of the Fit&Sober app can be established, patients with AUD can be provided with a valuable adjunct to traditional alcohol treatment that can be delivered in any setting and at any time, thereby improving the overall health and well-being of this population.

## Abbreviations

ADP: Alcohol and Drug Partial
AUD: alcohol use disorder
EOT: end of treatment
mHealth: mobile health
PA: physical activity
SUS: System Usability Scale
TLFB: Timeline Follow Back
